# Genomic and Phenotypic Analysis of COVID-19-Associated Pulmonary Aspergillosis Isolates of Aspergillus fumigatus

**DOI:** 10.1128/spectrum.00010-21

**Published:** 2021-06-09

**Authors:** Jacob L. Steenwyk, Matthew E. Mead, Patrícia Alves de Castro, Clara Valero, André Damasio, Renato A. C. dos Santos, Abigail L. Labella, Yuanning Li, Sonja L. Knowles, Huzefa A. Raja, Nicholas H. Oberlies, Xiaofan Zhou, Oliver A. Cornely, Frieder Fuchs, Philipp Koehler, Gustavo H. Goldman, Antonis Rokas

**Affiliations:** a Department of Biological Sciences, Vanderbilt University, Nashville, Tennessee, USA; b Faculdade de Ciências Farmacêuticas de Ribeirão Preto, Universidade de São Paulo, Ribeirão Preto, Brazil; c Institute of Biology, University of Campinas (UNICAMP), Campinas-SP, Brazil; d Experimental Medicine Research Cluster (EMRC), University of Campinas (UNICAMP), Campinas-SP, Brazil; e Department of Chemistry and Biochemistry, University of North Carolina at Greensboro, Greensboro, North Carolina, USA; f Guangdong Laboratory for Lingnan Modern Agriculture, Guangdong Province Key Laboratory of Microbial Signals and Disease Control, Integrative Microbiology Research Centre, South China Agricultural University, Guangzhou, China; g University of Cologne, Medical Faculty and University Hospital Cologne, Department I of Internal Medicine, Excellence Center for Medical Mycology (ECMM), Cologne, Germany; h University of Cologne, Cologne Excellence Cluster on Cellular Stress Responses in Aging-Associated Diseases (CECAD), Cologne, Germany; i ZKS Köln, Clinical Trials Centre Cologne, Cologne, Germany; j German Center for Infection Research (DZIF), Partner Site Bonn‐Cologne, Medical Faculty and University Hospital Cologne, University of Cologne, Cologne, Germany; k Faculty of Medicine, Institute for Medical Microbiology, Immunology and Hygiene, University of Cologne, Cologne, Germany; Broad Institute

**Keywords:** pathogenicity, coinfection, secondary infection, virulence factors, superinfection, acute respiratory distress syndrome, *Aspergillus*, aspergillosis, COVID-19

## Abstract

The ongoing global pandemic caused by the severe acute respiratory syndrome coronavirus 2 (SARS-CoV-2) is responsible for coronavirus disease 2019 (COVID-19), first described in Wuhan, China. A subset of COVID-19 patients has been reported to have acquired secondary infections by microbial pathogens, such as opportunistic fungal pathogens from the genus Aspergillus. To gain insight into COVID-19-associated pulmonary aspergillosis (CAPA), we analyzed the genomes and characterized the phenotypic profiles of four CAPA isolates of Aspergillus fumigatus obtained from patients treated in the area of North Rhine-Westphalia, Germany. By examining the mutational spectrum of single nucleotide polymorphisms, insertion-deletion polymorphisms, and copy number variants among 206 genes known to modulate A. fumigatus virulence, we found that CAPA isolate genomes do not exhibit significant differences from the genome of the Af293 reference strain. By examining a number of factors, including virulence in an invertebrate moth model, growth in the presence of osmotic, cell wall, and oxidative stressors, secondary metabolite biosynthesis, and the MIC of antifungal drugs, we found that CAPA isolates were generally, but not always, similar to A. fumigatus reference strains Af293 and CEA17. Notably, CAPA isolate D had more putative loss-of-function mutations in genes known to increase virulence when deleted. Moreover, CAPA isolate D was significantly more virulent than the other three CAPA isolates and the A. fumigatus reference strains Af293 and CEA17, but similarly virulent to two other clinical strains of A. fumigatus. These findings expand our understanding of the genomic and phenotypic characteristics of isolates that cause CAPA.

**IMPORTANCE** The global pandemic caused by severe acute respiratory syndrome coronavirus 2 (SARS-CoV-2), the etiological agent of coronavirus disease 2019 (COVID-19), has already killed millions of people. COVID-19 patient outcome can be further complicated by secondary infections, such as COVID-19-associated pulmonary aspergillosis (CAPA). CAPA is caused by Aspergillus fungal pathogens, but there is little information about the genomic and phenotypic characteristics of CAPA isolates. We conducted genome sequencing and extensive phenotyping of four CAPA isolates of Aspergillus fumigatus from Germany. We found that CAPA isolates were often, but not always, similar to other reference strains of A. fumigatus across 206 genetic determinants of infection-relevant phenotypes, including virulence. For example, CAPA isolate D was more virulent than other CAPA isolates and reference strains in an invertebrate model of fungal disease, but similarly virulent to two other clinical strains. These results expand our understanding of COVID-19-associated pulmonary aspergillosis.

## INTRODUCTION

On 11 March 2020, the World Health Organization declared the ongoing pandemic caused by SARS-CoV-2, which causes COVID-19, a global emergency (1). Similar to other viral infections, patients may be more susceptible to microbial secondary infections, which can complicate disease management strategies and result in adverse patient outcomes (2, 3). For example, approximately one quarter of patients infected with the H1N1 influenza virus during the 2009 pandemic were also infected with bacteria or fungi (4, 5). Among COVID-19 patients, one study found that ∼17% of individuals also have bacterial infections (6) and another that ∼40% of patients with severe COVID-19 pneumonia were also infected with filamentous fungi from the genus Aspergillus (7). A third study reported that ∼26% of patients with acute respiratory distress syndrome-associated COVID-19 were also infected with Aspergillus fumigatus and had high rates of mortality (8). Other studies from around the world have also reported high incidences of Aspergillus infections among patients with COVID-19 (9–12). Taken together, these findings have prompted some to suggest routine clinical testing for secondary infections of Aspergillus fungi among COVID-19 patients (13, 14). Despite the prevalence of microbial infections and their association with adverse patient outcomes, these secondary infections are only beginning to be understood.

**FIG 1:**
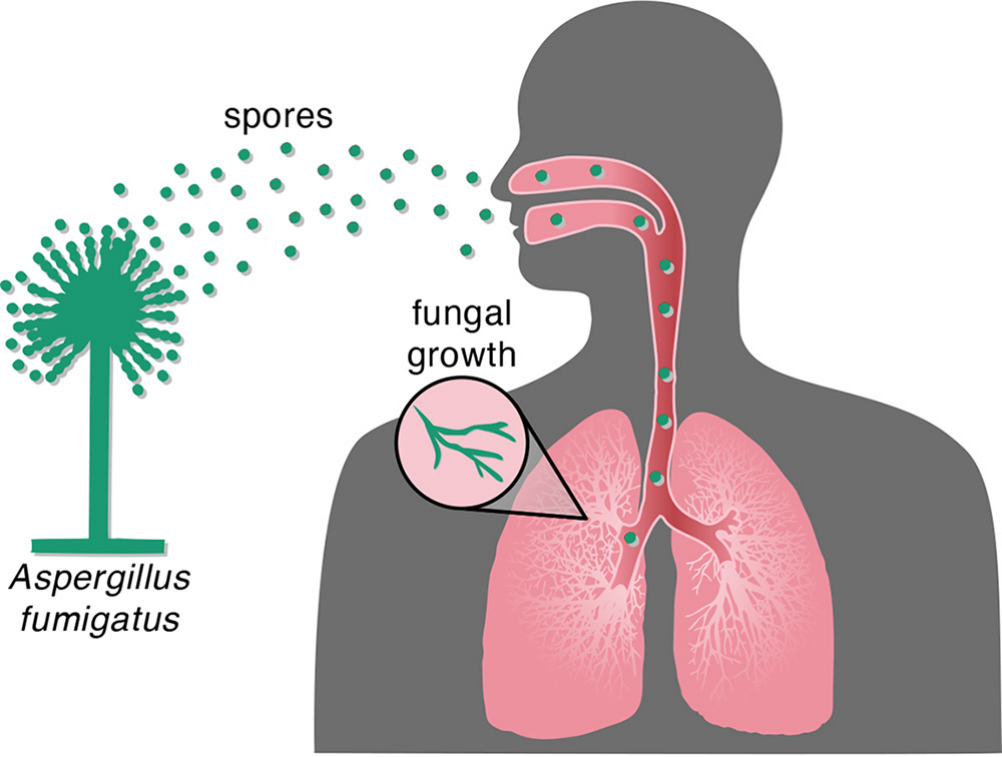
Inhalation of Aspergillus spores can result in fungal infection. Inhalation of Aspergillus spores from the environment can travel to the lung and then grow vegetatively and spread to other parts of the body.

Invasive pulmonary aspergillosis is caused by tissue infiltration of Aspergillus species after inhalation of their asexual spores (Fig. 1); more than 250,000 aspergillosis infections are estimated to occur annually and have high mortality rates (15). The major etiological agent of aspergillosis is A. fumigatus (16), although a few other Aspergillus species are also known to cause aspergillosis (17–20). Numerous factors are known to be associated with A. fumigatus pathogenicity, including its ability to grow at the human body temperature (37°C) and withstand oxidative stress (21–32). Disease management of A. fumigatus is further complicated by resistance to antifungal drugs among strains (33–36) and A. fumigatus strains have been shown to exhibit strain heterogeneity with respect to virulence and pathogenicity-associated traits (18, 32, 37–40). However, it remains unclear whether the genomic and pathogenicity-related phenotypic characteristics of CAPA isolates are similar to or distinct from those of previously studied clinical strains of A. fumigatus.

To address this question and gain insight into the pathobiology of A. fumigatus CAPA isolates, we examined the genomic and phenotypic characteristics of four CAPA isolates obtained from four critically ill patients of two different centers in Cologne, Germany (8) (Table 1). All patients were submitted to intensive care units due to moderate to severe respiratory distress syndrome (ARDS). Genome-scale phylogenetic (or phylogenomic) analyses revealed CAPA isolates formed a monophyletic group closely related to reference strains Af293 and A1163. Examination of the mutational spectra of 206 genes known to modulate virulence in A. fumigatus (which are here referred to as genetic determinants of virulence) revealed several putative loss-of-function (LOF) mutations. Notably, CAPA isolate D had the most putative LOF mutations among genes whose null mutants are known to increase virulence. The profiles of pathogenicity-related traits and of secondary metabolites of the CAPA isolates were similar to those of reference A. fumigatus strains Af293 and CEA17 or CEA10, which are parental strains of A1163 (41). One notable exception was that CAPA isolate D was significantly more virulent than other strains in an invertebrate model of disease, but on par with two other clinical strains of A. fumigatus. These results suggest that the genomes of A. fumigatus CAPA isolates contain nearly complete and intact repertoires of genetic determinants of virulence and have phenotypic profiles that are broadly expected for A. fumigatus clinical isolates. However, we did find evidence for genetic and phenotypic strain heterogeneity. These results suggest the CAPA isolates show similar phenotypic profiles as A. fumigatus clinical strains Af293 and A1163 and expand our understanding of CAPA.

**TABLE 1:** Metainformation and NCBI accessions for CAPA isolates

Isolate^*a*^	Patient identifier	Patient outcome	Patient age	Patient sex	Patient immunocompromising condition	Biological source of fungal isolate	Antifungal treatment^*c*^	Antiviral treatment^*b*^	NCBI BioSample/sequence read archive accessions
CAPA A	Patient from this study	Deceased	57	Male	None	Bronchial aspirate	Caspofungin (70/50 mg once daily)	Supportive only	SAMN16591136; SRR12949929
CAPA B	Patient no. 4	Deceased	73	Male	Inhalational steroids for medical history of chronic obstructive pulmonary disease	Bronchial aspirate	Voriconazole i.v. (6/4 mg/kg BW twice daily)	Supportive only	SAMN16591179; SRR12949928
CAPA C	Patient no. 3	Alive	54	Male	IV corticosteroid therapy 0.4 mg/kg/day, total of 13 days	Bronchoalveolar lavage fluid	Caspofungin (70/50 mg once daily) followed by i.v. voriconazole (6/4 mg/kg BW twice daily)	Hydroxychloroquine, darunavir and cobicistat at external hospital, in house changed to supportive only	SAMN16591190; SRR12949927
CAPA D	Patient no.1	Deceased	62	Female	Inhalational steroids for medical history of chronic obstructive pulmonary disease	Bronchoalveolar lavage fluid	Voriconazole i.v. (6/4 mg/kg BW twice daily)	Supportive only	SAMN16591200; SRR12949926

a Information on CAPA isolates B, C, and D is from reference 8, where the isolates were first described. CAPA isolate A is being reported for the first time.

b Supportive only antiviral treatment indicates no specific antiviral treatment was given.

c BW, body weight; i.v., intravenous; kg, kilogram; mg, milligram.

## RESULTS AND DISCUSSION

### CAPA isolates belong to A. fumigatus and are closely related to reference strains Af293 and A1163.

**FIG 2:**
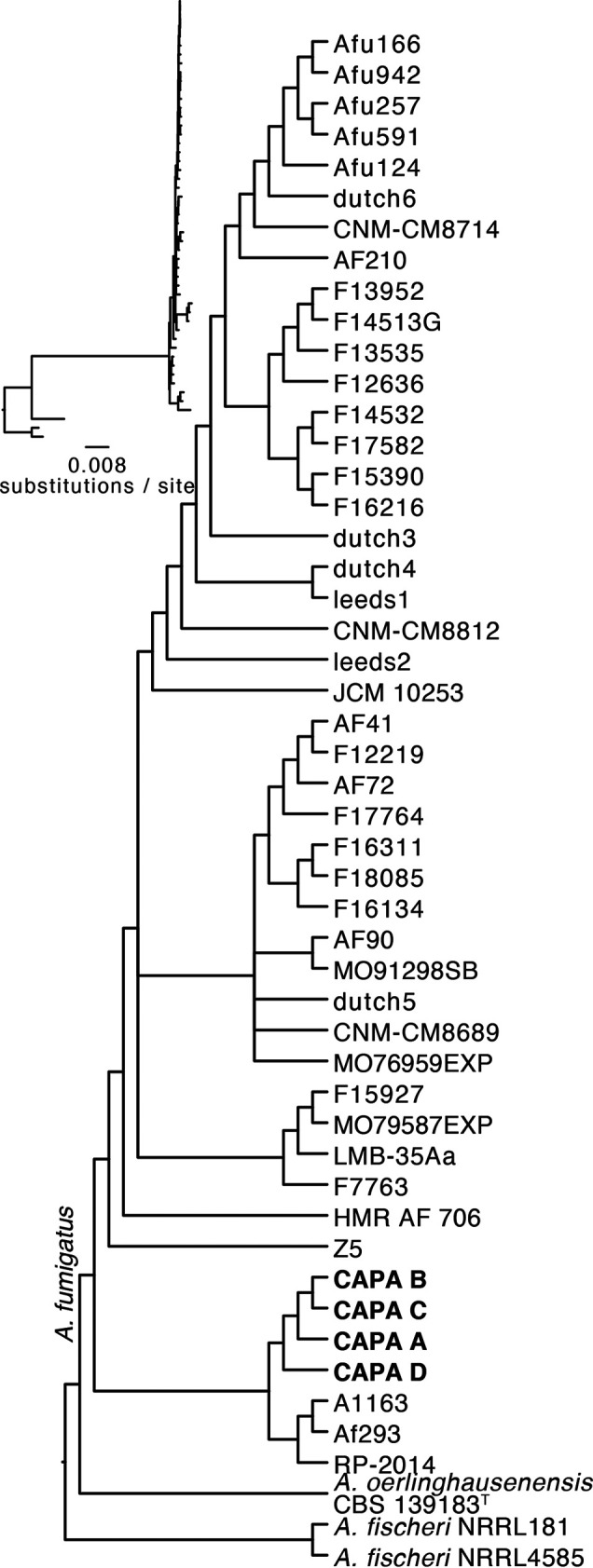
Phylogenomics confirms that COVID-19-associated pulmonary aspergillosis (CAPA) isolates are Aspergillus fumigatus. Phylogenomic analysis of a concatenated matrix of 4,525 single-copy orthologous groups genes (sites: 7,133,367) confirmed CAPA isolates are A. fumigatus. Furthermore, CAPA isolates are closely related to reference strains A1163 and Af293. Bipartitions with less than 85% ultrafast bootstrap approximation support were collapsed.

To confirm that the CAPA isolates belong to A. fumigatus, we sequenced, assembled, and annotated their genomes (Table S1 in the supplemental material; see figshare, 10.6084/m9.figshare.13118549). Phylogenetic analyses conducted using *tef1* (Fig. S1) and calmodulin (Fig. S2) sequences suggested that all CAPA isolates are A. fumigatus. Phylogenomic analysis using 50 Aspergillus genomes (the four CAPA isolates, 43 A. fumigatus genomes that span the known diversity of the species, including strains Af293 and A1163 (18, 29, 42–47), Aspergillus fischeri strains NRRL 181 and NRRL 4585, and Aspergillus oerlinghausenensis strain CBS 139183^T^) (40, 45) confirmed that all CAPA isolates are A. fumigatus (Fig. 2). Phylogenomic analyses also revealed the CAPA isolates formed a monophyletic group closely related to reference strains Af293 and A1163. CAPA isolates are inferred to be closely related, which may be due to the fact they are all from the same geographic area.

### CAPA isolate genomes contain polymorphisms in genetic determinants of virulence and biosynthetic gene clusters.

An extensive literature and database search identified 206 genetic determinants of virulence (File S1) (23, 48–51). We define genetic determinants of virulence as genes that alter virulence in an animal model of disease when deleted or that are required for biosynthesis of secondary metabolites known to affect virulence. This definition resulted in a list of genes distinct from those previously published, which include genes that contribute to allergy-related phenotypes and genes that are computationally predicted to contribute to virulence but have yet to be validated in an animal model of fungal disease (52–55).

**FIG 3:**
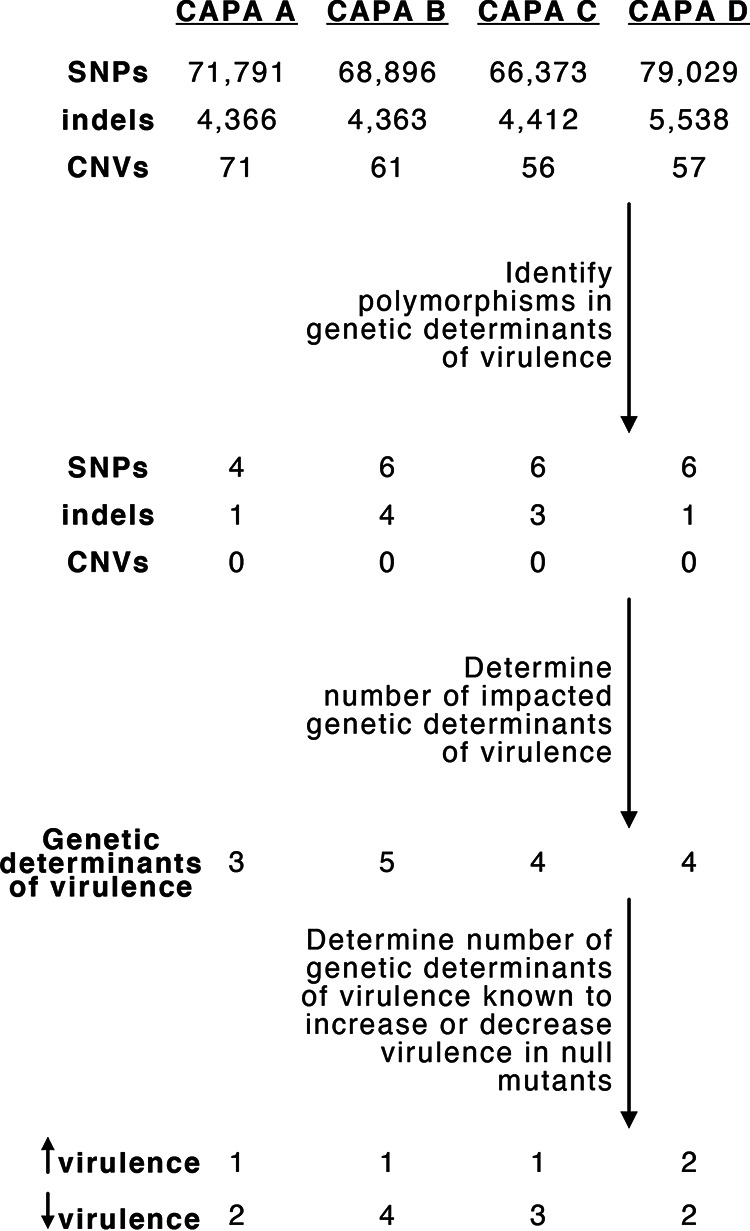
Mutational spectra among genetic determinants of virulence. Genome-wide SNPs, indels, and CN variants were filtered for those present in genetic determinants of virulence. Then, the number of genetic determinants of virulence with high-impact polymorphisms was identified. The number known to increase or decrease virulence in null mutants was determined thereafter.

To determine if the 206 genetic determinants of virulence are conserved in CAPA isolates, we conducted sequence similarity searches of gene sequences in the genomes of the CAPA isolates. We found that all 206 genes were present in the genomes of the CAPA isolates. Furthermore, none of the 206 genetic determinants of virulence showed any copy number variation among CAPA isolates. Examination of single nucleotide polymorphisms (SNPs) and insertion/deletion (indel) polymorphisms coupled with variant effect prediction in these 206 genes (Fig. 3; File S2) showed that all CAPA isolates shared multiple polymorphisms resulting in early stop codons or frameshift mutations suggestive of loss of function (LOF) in *NRPS8* (AFUA_5G12730), a nonribosomal peptide synthetase gene that encodes an unknown secondary metabolite (42). LOF mutations in *NRPS8* are known to result in increased virulence in a mouse model of disease (56). Putative LOF mutations were also observed in genes whose null mutants decreased virulence. For example, all CAPA isolates shared the same SNPs resulting in early stop codons that likely result in LOF or partial LOF in *pptA* (AFUA_2G08590), a putative 4′-phosphopantetheinyl transferase, whose deletion results in reduced virulence in a mouse model of disease (57). In light of the close evolutionary relationships among CAPA isolates, we hypothesize that these shared mutations likely occurred in the genome of their most recent common ancestor.

In addition to shared polymorphisms, analyses of CAPA isolate genomes also revealed isolate-specific polymorphisms affecting genetic determinants of virulence (File S2). For example, SNPs resulting in early stop codons, which likely lead to LOF, were observed in *CYP5081A1* (AFUA_4G14780), a putative cytochrome P450 monooxygenase, in CAPA isolates B and C. *CYP5081A1* LOF is associated with reduced virulence of A. fumigatus (58). Other SNPs were found only in single isolates. CAPA isolate B has a frameshift mutation in a putative fatty acid oxygenase (AFUA_4G00180). CAPA isolate D has a mutation resulting in the loss of the start codon in *fleA* (AFUA_5G14740), a gene that encodes an l-fucose-specific lectin. Mice infected with *FLEA* null mutants have more severe pneumonia and invasive aspergillosis than wild-type strains. *FLEA* null mutants cause more severe disease because FleA binds to macrophages and therefore is critical for host recognition, clearance, and macrophage killing (59). The only evidence of pseudogenization among the genetic determinants of virulence in the reference strains was observed in *mybA* (AFUA_3G07070), a transcription factor involved in conidiation and conidial viability (60), in strain A1163. *MybA* null mutants have reduced virulence compared to wild-type strains (60).

Examination of three additional clinical strains of A. fumigatus (IFM61407, CN-CM7555, and Afs35) revealed that CAPA isolates shared some, but not all, polymorphisms present in the 206 genetic determinants of virulence. For example, similar putative LOF mutations were observed in *NRPS8* (AFUA_5G12730). Polymorphisms that were not shared between CAPA isolates and the three clinical strains include the loss of a stop codon in *aspA*, a septin (61), in strain Afs35; an early stop codon in *noc3*, a nuclear export protein (62), in strain CN-CM7555; and a lost stop codon in *cat2*, a bifunctional catalase-peroxidase (63), in strain IFM61407. A complete list of high-impact polymorphisms in the three clinical strains is available in File S2.

**FIG 4:**
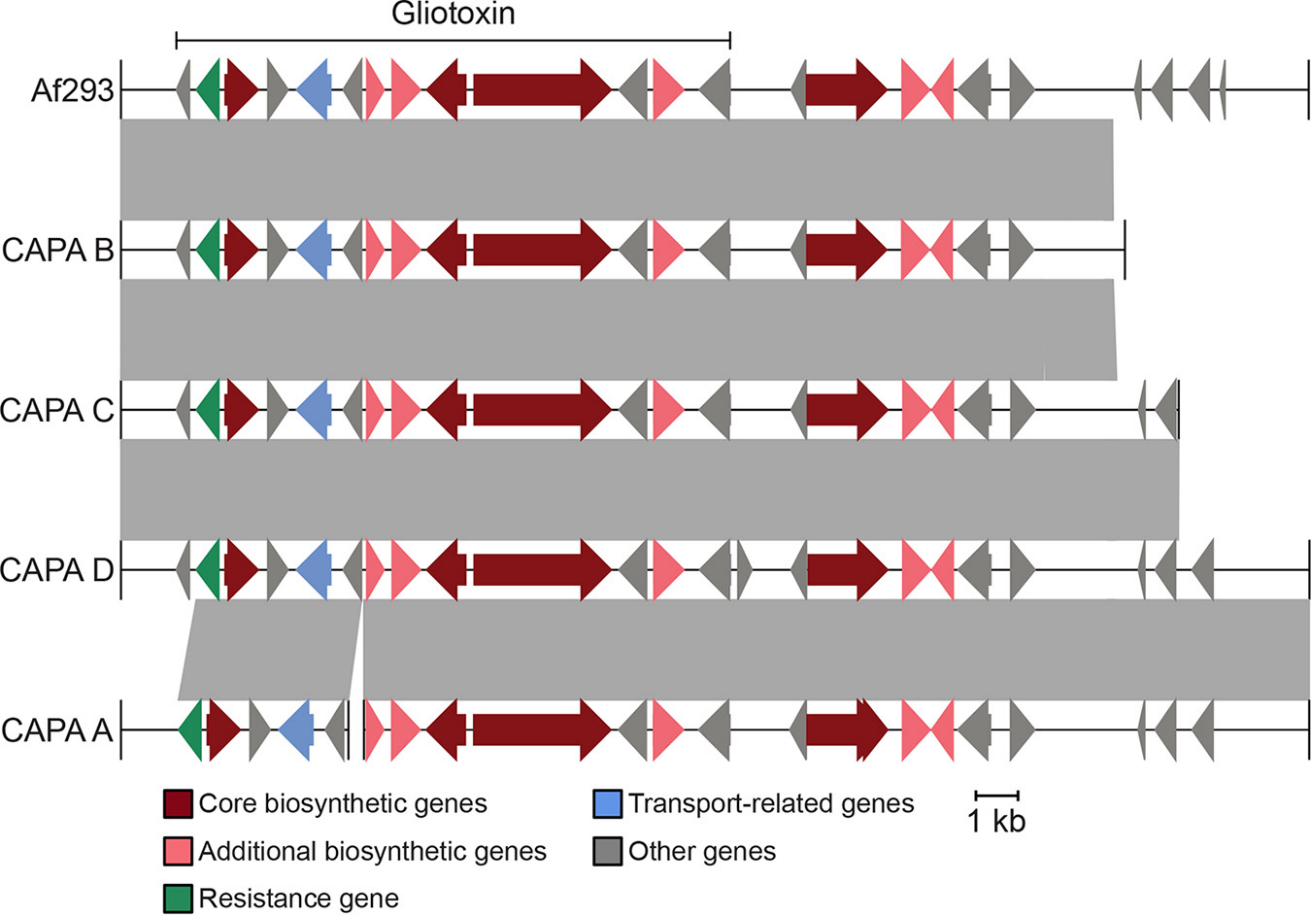
COVID-19-associated pulmonary aspergillosis (CAPA) isolates of Aspergillus fumigatus have biosynthetic gene clusters (BGCs) that encode the toxic small molecule gliotoxin. Gliotoxin is known to contribute to virulence of A. fumigatus. The genomes of CAPA isolates of A. fumigatus contain biosynthetic gene clusters known to encode gliotoxin. Note, the BGC of CAPA isolate A was split between two contigs and, therefore, the BGC is hypothesized to be present.

Examination of the presence of biosynthetic gene clusters (BGCs) revealed that all CAPA isolates had BGCs that encode secondary metabolites known to modulate host biology (Table 2). For example, all CAPA isolates had BGCs encoding the toxic secondary metabolite gliotoxin (Fig. 4). Other intact BGCs in the genomes of the CAPA isolates include fumitremorgin, trypacidin, pseurotin, and fumagillin, which are known to modulate host biology (64–66); for example, fumagillin is known to inhibit neutrophil function (67, 68). More broadly, all CAPA isolates had similar numbers and classes of BGCs (Fig. S3).

**TABLE 2:** Biosynthetic gene clusters that produce secondary metabolites implicated in modulating host biology in A. fumigatus

Metabolite	Function	Reference(s)	Evidence of biosynthetic gene cluster/secondary metabolite^*a*^			
			CAPA A	CAPA B	CAPA C	CAPA D
Gliotoxin	Inhibits host immune response	72	+/+	+/+	+/+	+/+
Fumitremorgin	Inhibits the breast cancer resistance protein	64	+/+	+/+	+/+	+/+
Trypacidin	Damages lung cell tissues	65	+/−	+/−	+/−	+/−
Pseurotin	Inhibits immunoglobulin E	66	+/+	+/+	+/+	+/−
Fumagillin	Inhibits neutrophil function	67, 68	+/+	+/+	+/+	+/+

a The symbols + and − indicate the presence and absence of a BGC and secondary metabolite, respectively. For example, +/− indicates the presence of the BGC but no evidence of secondary metabolite production. Evidence of robust secondary metabolite production is defined as evidence of the secondary metabolite across all three replicates. (See Fig. S6 in the supplemental material for relative abundance of secondary metabolite production.)

In summary, we found that CAPA isolates were closely related to one another and had largely intact genetic determinants of virulence and BGCs. However, we observed strain-specific polymorphisms in known genetic determinants of virulence in CAPA isolate genomes, which raises the hypothesis that CAPA isolates differ in their virulence profiles.

### CAPA isolates display strain heterogeneity in virulence and in a few virulence-related traits.

**FIG 5:**
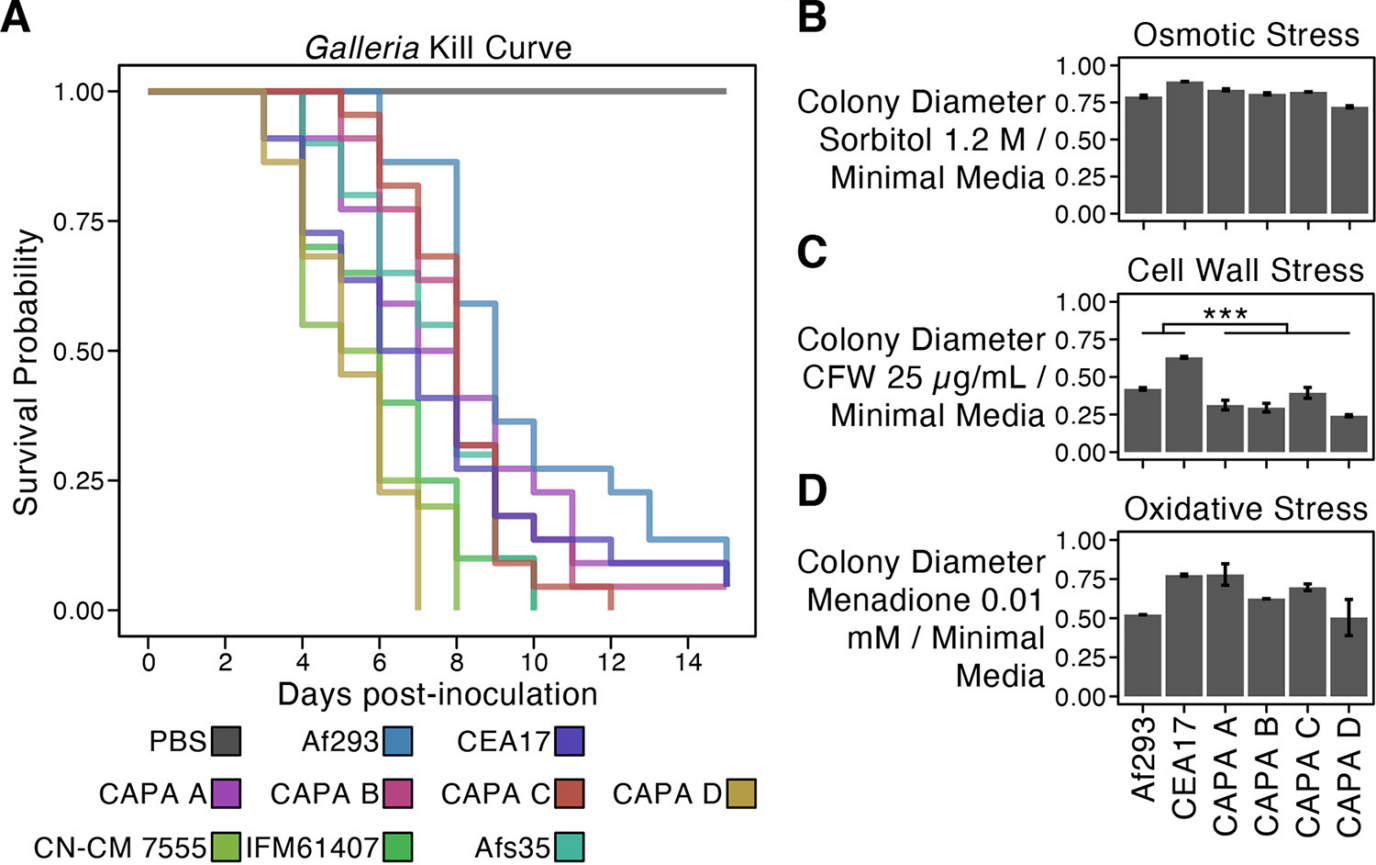
Strain heterogeneity among COVID-19-associated pulmonary aspergillosis (CAPA) isolates of Aspergillus fumigatus. (A) The virulence of the CAPA isolates, reference strains Af293 and CEA17, and clinical strains Afs35, CN-CM7555, and IFM61407 significantly varied in the *Galleria* moth model of disease (*P* < 0.001; log-rank test; ≥20 replicates per strain). Pairwise examinations revealed CAPA isolate D was significantly more virulent than all other strains (Benjamini-Hochberg adjusted *P* < 0.01 when comparing CAPA isolate D to another isolate; log-rank test) with the exception of clinical strains IFM61407 and CN-CM 7555 (*P* = 0.085 and *P* = 0.386, respectively). Growth of CAPA isolates and references strains Af293 and CEA17 in the presence of osmotic (B), cell wall (C), and oxidative stressors (D). Growth differences between CAPA isolates and reference strains Af293 and CEA17 were observed across all growth conditions (*P* < 0.001; multifactor ANOVA). Pairwise differences were assessed using the *post hoc* Tukey’s honestly significant difference (HSD) test and were only observed for growth in the presence of CFW at 25 μg/ml (*P* < 0.001; Tukey HSD test) in which the CAPA isolates did not grow as well as the reference isolates. To correct for strain heterogeneity in growth rates, radial growth in centimeters in the presence of stressors was divided by radial growth in centimeters in the absence of the stressor (MM only). Abbreviations of cell wall stressors are as follows: CFW, calcofluor white; CR, Congo red; CSP, caspofungin. Growth in the presence of other stressors is summarized in Fig. S4. Error bars in panels B to D represent the average of ± one standard deviation across three replicates.

Examination of virulence and virulence-related traits revealed the CAPA isolates often, but not always, had similar phenotypic profiles compared to reference A. fumigatus strains Af293 and a CEA17 Δ*akuB*^KU80^ pyrG^+^ derivative of CEA17 *akuB*^KU80+^, pyrG^−^ (which is here referred to as CEA17 for simplicity [41]). For example, virulence in the *Galleria* moth model of fungal disease revealed strain heterogeneity among CAPA isolates Af293, CEA17, and a panel of three clinical strains of A. fumigatus, namely, Afs35, IFM61407, and CN-CM7555 (41, 69, 70) (*P* < 0.001; log-rank test; Fig. 5A). Pairwise examination revealed the observed strain heterogeneity was primarily driven by CAPA isolate D, which was significantly more virulent than all other CAPA isolates, reference strains Af293 and CEA17, and clinical strain Afs35 (Benjamini-Hochberg adjusted *P* value < 0.05 when comparing CAPA isolate D to another isolate; log-rank test; File S3). However, the virulence of the CAPA isolate D was on par to those of clinical strains IFM61407 and CN-CM 7555 (*P* = 0.085 and *P* = 0.386, respectively) (Fig. 5A). These results reveal the CAPA isolates have generally similar virulence profiles compared to the reference strains Af293 and CEA17 with the exception of the more virulent CAPA isolate D. Furthermore, the virulence profiles of all CAPA isolates are within the known range of A. fumigatus clinical strains. Determining the association between virulence and the genetic polymorphisms described in the section above, in addition to other polymorphisms identified in this study (Fig. 3), is an important future direction.

Examination of growth in the presence of osmotic, cell wall, and oxidative stressors revealed the phenotypic profiles of CAPA isolates were similar to the profiles of Af293 and CEA17 strains (Fig. 5B to D and Fig. S4). The sole exception was growth in the presence of calcofluor white, where we observed that the CAPA isolates were more sensitive than reference strains Af293 and CEA17 (*P* < 0.001; Tukey’s honestly significant difference test; Fig. 5C). Last, antifungal drug susceptibility profiles for amphotericin B, voriconazole, itraconazole, and posaconazole were similar between the CAPA isolates and reference strains Af293 and CEA17 (Table 3). Following the guidelines of the Clinical and Laboratory Standards Institute (71), the CAPA isolates are not considered multidrug resistant.

**TABLE 3:** Antifungal drug susceptibility (MICs in μg/ml) of CAPA clinical isolates grown in minimal medium

Drug	Af293	CEA17	CAPA A	CAPA B	CAPA C	CAPA D
Amphotericin B	2	2	2–4	2	2	2
Voriconazole	1	0.25–0.50	0.5	0.5	0.25–0.50	0.5
Itraconazole	0.5	0.5	0.5	0.5	0.5	0.5
Posaconazole	1	1	1	1	1	1

Secondary metabolites can impact host biology and virulence (30, 72). Examination of secondary metabolite production revealed strain heterogeneity among CAPA isolates and reference strains Af293 and CEA10, a pyrG^+^ and *akuB*^KU80+^ strain that CEA17 is derived from (41). For example, principal-component analysis of chromatogram features revealed that CAPA isolate A was substantially different from other CAPA isolates along the first and second principal components, which capture 82.47% of the total variance, whereas the CAPA isolate D was substantially different from other CAPA isolates along the second and third principal components, which capture 38.64% of total variance (Fig. S5). Examination of the loadings plot, which identifies the individual secondary metabolites that contribute to the observed variation across strains, revealed gliotoxin and fumitremorgin as the largest contributors (Fig. S6). Measurement of relative abundance of biosynthesized gliotoxin and fumitremorgin, two secondary metabolites known to modulate host biology (30), showed the largest amount of fumitremorgin was biosynthesized by CAPA isolate A, and the largest amount of gliotoxin was biosynthesized by the Af293 strain, followed by CAPA isolate C (Fig. S6; Table 2).

In summary, we found the CAPA isolates have similar phenotypic profiles, with the exception of growth in the presence of calcofluor white and secondary metabolite biosynthesis, compared to reference strains, and virulence on par with the known range of A. fumigatus clinical strains.

In conclusion, the effects of secondary fungal infections in COVID-19 patients are only beginning to be understood. Our results revealed that CAPA isolates are generally, but not always, similar to A. fumigatus clinical reference strains. Notably, CAPA isolate D was significantly more virulent than the other three CAPA isolates and two reference strains examined, but on par with other clinical strains. Taken together, these results are important to consider in the management of fungal infections among patients with COVID-19, especially those infected with A. fumigatus, and broaden our understanding of CAPA.

## MATERIALS AND METHODS

### Patient information and ethics approval.

Patients were included into the FungiScope global registry for emerging invasive fungal infections (www.ClinicalTrials.gov, NCT 01731353). The clinical trial is approved by the Ethics Committee of the University of Cologne, Cologne, Germany (study ID: 05-102) (73). Since 2019, patients with invasive aspergillosis were also included.

### DNA quality control, library preparation, and sequencing.

Sample DNA concentration was measured by Qubit fluorometer and DNA integrity and purity by agarose gel electrophoresis. For each sample, 1 to 1.5 μg of genomic DNA was randomly fragmented by Covaris and fragments with average sizes of 200 to 400 bp were selected by Agencourt AMPure XP-Medium kit. The selected fragments were end-repaired, 3′ adenylated, ligated to adapters, and amplified by PCR. Double-stranded PCR products were recovered by the AxyPrep Mag PCR cleanup kit, and then heat denatured and circularized by using the splint oligonucleotide sequence. The single-strand circlular DNA (ssCir DNA) products were formatted as the final library and went through further QC procedures. The libraries were sequenced on the MGISEQ2000 platform.

### Genome assembly and annotations.

Short-read sequencing data of each sample were assembled using MaSuRCA, v3.4.1 (74). Each *de novo* genome assembly was annotated using the MAKER genome annotation pipeline, v2.31.11 (75), which integrates three *ab initio* gene predictors: AUGUSTUS, v3.3.3 (76), GeneMark-ES, v4.59 (77), and SNAP, v2013-11-29 (78). Fungal protein sequences in the Swiss-Prot database (release 2020_02) were used as homology evidence for the genome annotation. The MAKER annotation process occurs in an iterative manner as described previously (79). In brief, for each genome, repeats were first soft-masked using RepeatMasker v4.1.0 (http://www.repeatmasker.org) with the library Repbase library release-20181026 and the “-species” parameter set to “Aspergillus fumigatus.” GeneMark-ES was then trained on the masked genome sequence using the self-training option (“–ES”) and the branch model algorithm (“–fungus”), which is optimal for fungal genome annotation. On the other hand, an initial MAKER analysis was carried out where gene annotations were generated directly from homology evidence, and the resulting gene models were used to train both AUGUSTUS and SNAP. Once trained, the *ab initio* predictors were used together with homology evidence to conduct a first round of full MAKER analysis. Resulting gene models supported by homology evidence were used to retrain AUGUSTUS and SNAP. A second round of MAKER analysis was conducted using the newly trained AUGUSTUS and SNAP parameters, and once again the resulting gene models with homology supports were used to retrain AUGUSTUS and SNAP. Finally, a third round of MAKER analysis was performed using the new AUGUSTUS and SNAP parameters to generate the final set of annotations for the genome. The completeness of *de novo* genome assemblies and *ab initio* gene predictions was assessed using BUSCO, v4.1.2 (80) using 4,191 preselected “nearly” universally single-copy orthologous genes from the *Eurotiales* database (eurotials_odb10.2019-11-20) in OrthoDB, v10.1 (81).

### Polymorphism identification.

To characterize and examine the putative impact of polymorphisms in the genomes of the CAPA isolates, we identified single nucleotide polymorphisms (SNPs), insertion-deletion polymorphisms (indels), and copy number (CN) polymorphisms. To do so, reads were first quality-trimmed and mapped to the genome of A. fumigatus Af293 (RefSeq assembly accession: GCF_000002655.1) following a previously established protocol (82). Specifically, reads were first quality-trimmed with Trimmomatic v0.36 (83) using the following parameters: leading, 10; trailing, 10; slidingwindow, 4:20; minlen, 50. The resulting quality-trimmed reads were mapped to the A. fumigatus Af293 genome using the Burrows-Wheeler Aligner (BWA) v0.7.17 (84) with the mem parameter. Thereafter, mapped reads were converted to a sorted bam and mpileup format for polymorphism identification using SAMtools v.1.3.1 (85).

To identify SNPs and indels, mpileup files were used as input into VarScan v2.3.9 (86), with the mpileup2snp and mpileup2indel functions, respectively. To ensure only confident SNPs and indels were identified, a Fisher’s exact test *P* value threshold of 0.05 and minimum variant allele frequency of 0.75 were used. The resulting Variant Call Format files were used as input to snpEff v.4.3t (87), which predicted their functional impacts on gene function as high, moderate, or low. To identify CN variants, the sorted bam files were used as input into Control-FREEC v9.1 (88, 89). The coefficientOfVariation parameter was set to 0.062 and window size was automatically determined by Control-FREEC. To ensure high confidence in CN variant identification, a *P* value threshold of 0.05 was used for both the Wilcoxon rank sum and Kolmogorov Smirnov tests.

To identify evidence of putative pseudogenization between reference strains A1163 and Af293, we used a previously established approach (19, 90). More specifically, we compared lengths of gene pairs as a proxy for pseudogenization. A gene was considered a putative pseudogene in one of the strains if the gene was 70% the length of its reciprocal best blast hit in the other strain.

### Maximum likelihood molecular phylogenetics.

To taxonomically identify the species of Aspergillus sequenced, we conducted molecular phylogenetic analysis of two different loci and two different data sets. In the first analysis, the nucleotide sequence of the alpha subunit of translation elongation factor EF-1, *tef1* (NCBI accession: XM_745295.2), from the genome of A. fumigatus Af293 was used to extract other fungal *tef1* sequences from NCBI’s fungal nucleotide reference sequence database (downloaded July 2020) using the blastn function from NCBI’s BLAST+, v2.3.0 (91). *Tef1* sequences were extracted from the CAPA isolates by identifying their best BLAST hit. Sequences from the top 100 best BLAST hits in the fungal nucleotide reference sequence database and the four *tef1* sequences from the CAPA isolates were aligned using MAFFT v7.402 (92) using previously described parameters (93) with slight modifications. Specifically, the following parameters were used: –op 1.0 –maxiterate 1000 –retree 1 –genafpair. The resulting alignment was trimmed using ClipKIT v0.1 (19) with default “gappy” mode. The trimmed alignment was then used to infer the evolutionary history of *tef1* sequences using IQ-TREE2 (94). The best-fitting substitution model—TIM3 with empirical base frequencies, allowing for a proportion of invariable sites, and a discrete Gamma model (95, 96) with four rate categories (TIM3+F+I+G4)—was determined using the Bayesian information criterion. In the second analysis, the same process was used to conduct molecular phylogenetic analysis using calmodulin nucleotide sequences from Aspergillus section *Fumigati* species and Aspergillus clavatus, an outgroup taxon, using sequences from NCBI that were made available elsewhere (97). For calmodulin sequences, the best-fitting substitution model was TNe (98) with a discrete Gamma model with four rate categories (TNe+G4). Bipartition support was assessed using 5,000 ultrafast bootstrap support approximations (99).

To determine which strains of A. fumigatus the CAPA isolates were most similar to, we conducted phylogenomic analyses using the 50 Aspergillus proteomes. To do so, we first identified orthologous groups of genes across all 50 Aspergillus strains using OrthoFinder 2.3.8 (100). OrthoFinder takes as input the proteome sequence files from multiple genomes and conducts all-versus-all sequence similarity searches using DIAMOND v0.9.24.125 (101). Our input included 50 total proteomes, where 47 were A. fumigatus, two were A. fischeri, and one was *A. oerlinghausenensis* (40, 42, 45). OrthoFinder then clusters sequences into orthologous groups of genes using the graph-based Markov Clustering Algorithm (102). To maximize the number of single-copy orthologous groups of genes found across all input genomes, clustering granularity was explored by running 41 iterations of OrthoFinder that differed in their inflation parameter. Specifically, iterations of OrthoFinder inflation parameters were set to 1.0 to 5.0 with a step of 0.1. The lowest number of single-copy orthologous groups of genes was 3,399 when using an inflation parameter of 1.0; the highest number was 4,525 when using inflation parameter values of 3.8 and 4.1. We used the groups inferred using an inflation parameter of 3.8.

Next, we built the phylogenomic data matrix and reconstructed evolutionary relationships among the 50 Aspergillus genomes. To do so, the protein sequences from 4,525 single-copy orthologous groups of genes were aligned using MAFFT v7.402 (92), with the following parameters: –bl 62 –op 1.0 –maxiterate 1000 –retree 1 –genafpair. Next, nucleotide sequences were threaded onto the protein alignments using function thread_dna in PhyKIT v0.0.1 (103). The resulting codon-based alignments were then trimmed using ClipKIT v0.1 (104), using the gappy mode. The resulting aligned and trimmed alignments were then concatenated into a single matrix with 7,133,367 sites using the PhyKIT function create_concat. To reconstruct the evolutionary history of the 50 Aspergillus genomes, a single best-fitting model of sequence substitution and rate heterogeneity was estimated across the entire matrix using IQ-TREE2 v.2.0.6 (94). The best-fitting model was determined to be a general time reversible model with empirical base frequencies and invariable sites with a discrete Gamma model with four rate categories (GTR+F+I+G4) (96, 105–107) using the Bayesian information criterion. During tree search, the number of candidate trees maintained during maximum likelihood tree search was increased from five to ten. Five independent searches were conducted and the tree with the best log-likelihood score was chosen as the “best” phylogeny. Bipartition support was evaluated using 5,000 ultrafast bootstrap approximations (99).

### Biosynthetic gene cluster prediction.

To predict BGCs in the genomes of A. fumigatus strains Af293 and the CAPA isolates, gene boundaries inferred by MAKER were used as input into antiSMASH v4.1.0 (108). Using a previously published list of genes known to encode BGCs in the genome of A. fumigatus Af293 (42), BLAST-based searches using an expectation value threshold of 1 × 10^−10^ were used to identify BGCs implicated in modulating host biology using NCBI’s BLAST+ v2.3.0 (91). Among predicted BGCs that did not match the previously published list, we further examined their evolutionary history if at least 50% of genes showed similarity to species outside the genus Aspergillus, which is information provided in the antiSMASH output. Using these criteria, no evidence suggestive of horizontally acquired BGCs from distant relatives was detected.

### Characterization of biosynthesized secondary metabolites.

**(i) General experimental procedures.** HRESIMS experiments utilized a Thermo LTQ Orbitrap XL mass spectrometer equipped with an electrospray ionization source. A Waters Acquity UPLC (Waters Corp.) was utilized using a BEH C_18_ column (1.7 μm; 50 mm × 2.1 mm) set to a temperature of 40°C and a flow rate of 0.3 ml/min. The mobile phase consisted of a linear gradient of CH_3_CN-H_2_O (both acidified with 0.1% formic acid), starting at 15% CH_3_CN and increasing linearly to 100% CH_3_CN over 8 min, with a 1.5 min hold before returning to the starting condition.

**(ii) Growth and extraction of fungal cultures.** To identify the chemical differences between the various A. fumigatus strains and isolates (Af293, CEA10, CAPA isolates A, B, C, and D), they were grown in a clinically relevant growth condition (37°C) and extracted for chemometric analysis. Czapek Dox agar (Sigma-Aldrich) petri plates were inoculated with the asexual spores of each strain in biological triplicates. Subsequently, the plates were incubated at 37°C in the dark for 3 days. The cultures were extracted by chopping and transferring the agar to 20-ml scintillation vials, adding 10 ml of acetone, thoroughly shaking, and then letting the samples sit for 4 h. Last, the cultures were filtered and evaporated to dryness under nitrogen gas.

**(iii) Metabolomics analyses.** Principal-component analysis (PCA) was performed on the ultraperformance liquid chromatography-mass spectrometry (UPLC-MS) data. Untargeted UPLC-MS data sets for each sample were individually aligned, filtered, and analyzed using MZmine 2.20 software (https://sourceforge.net/projects/mzmine/) (109). Peak detection was achieved using the following parameters: noise level (absolute value), 1.5 × 10^5^; minimum peak duration, 0.05 min; *m/z* variation tolerance, 0.05; and *m/z* intensity variation, 20%. Peak list filtering and retention time alignment algorithms were used to refine peak detection. The join algorithm integrated all sample profiles into a data matrix using the following parameters: *m/z* and retention time balance set at 10.0 each, *m/z* tolerance set at 0.001, and RT tolerance set at 0.5 min. The resulting data matrix was exported to Excel (Microsoft) for analysis as a set of *m/z*-retention time pairs with individual peak areas detected in quadruplicate analyses. Samples that did not possess detectable quantities of a given marker ion were assigned a peak area of zero to maintain the same number of variables for all sample sets. Ions that did not elute between 2 and 8 min and/or had an *m/z* ratio less than 100 or greater than 1,200 Da were removed from analysis. Relative standard deviation was used to quantify variance between the technical replicate injections, which may differ slightly based on instrument variance. A cutoff of 1.0 was used at any given *m/z*-retention time pair across the technical replicate injections of one biological replicate and, if the variance was greater than the cutoff, it was assigned a peak area of zero (110). Final chemometric analysis, including data filtering and PCA, was conducted using Python. The PCA plots were generated using data from the averaged biological replicates from the petri dish cultures. Each biological replicate was plotted using averaged peak areas obtained across four replicate injections (technical replicates). The principal components (PC) were generated and processed via Scikit Learn decomposition and Pandas v0.25.3, Python libraries. The PCA data were plotted using Altair v4.1.0, Python graphing libraries. These data were converted into a dataframe via Pandas, and the PCs were created from the dataframe using Scikit Learn decomposition. The PCA scores and loadings plots were then plotted using the PCs dataframe that was generated from Scikit Learn.

### Infection of Galleria mellonella.

Survival curves (*n* ≥ 20/strain) were generated for Galleria mellonella infected with CAPA isolates A, B, C, and D. Phosphate-buffered saline (PBS) without asexual spores (conidia) was administered as a negative control. A log-rank test was used to examine strain heterogeneity, followed by pairwise comparisons with the Benjamini-Hochberg multitest correction (111). All the selected larvae of Galleria mellonella were in the final (sixth) instar larval stage of development, weighing 275 to 330 mg. Fresh conidia from each strain were harvested from minimal medium (MM) plates in PBS solution and filtered through a Miracloth (Calbiochem). For each strain, the spores were counted using a hemocytometer and the stock suspension was done at 2 × 10^8^ conidia/ml. The viability of the administered inoculum was determined by plating a serial dilution of the conidia on MM medium at 37°C. A total of 5 μl (1 × 10^6^ conidia/larva) from each stock suspension was inoculated per larva. The control group was composed of larvae inoculated with 5 μl of PBS to observe the killing due to physical trauma. The inoculum was performed using Hamilton syringe (7000.5KH) via the last left proleg. After infection, the larvae were maintained in petri dishes at 37°C in the dark and were scored daily. Larvae were considered dead by presenting the absence of movement in response to touch.

### Growth assays.

To examine growth conditions of the CAPA isolates and reference strains Af293 and CEA17, plates were inoculated with 10^4^ spores per strain and allowed to grow for 5 days on solid MM or MM supplemented with various concentrations of osmotic (sorbitol, NaCl), cell wall (Congo red, calcofluor white, and caspofungin), and oxidative stress agents (menadione and t-butyl) at 37°C. MM had 1% (wt/vol) glucose, original high-nitrate salts, trace elements, and a pH of 6.5; trace elements, vitamins, and nitrate salt compositions follow standards described elsewhere (112). To correct for strain heterogeneity in growth rates, radial growth in centimeters in the presence of stressors was divided by radial growth in centimeters in the absence of the stressor.

To determine the MICs of antifungal drugs in the CAPA isolates and reference strains Af293 and CEA17, strains were grown in 96-well plates at a concentration of 10^4^ spores/well in 200 μl of RPMI 1640 supplemented with increasing concentrations of amphotericin B, voriconazole, itraconazole, and posaconazole, according to the protocol elaborated by the Clinical and Laboratory Standards Institute (71). The MIC was defined as the lowest concentration of drugs that visually inhibited 100% fungal growth. Three independent experiments were carried out for each antifungal drug.

### Data availability.

Newly sequenced genomes assemblies, annotations, and raw short reads have been deposited to NCBI’s GenBank database under BioProject accession PRJNA673120.

Supplementary data, including tables, figures, and files, additional copies of genome assemblies, annotations, and gene coordinates, raw data, including the genome assembly and annotations of all analyzed Aspergillus genomes, the aligned and trimmed phylogenetic and phylogenomic data matrices, polymorphisms identified in the present project, and predicted BGCs have been uploaded to figshare (10.6084/m9.figshare.13118549).

## References

[1] Sohrabi C, Alsafi Z, O'Neill N, Khan M, Kerwan A, Al-Jabir A, Iosifidis C, Agha R. 2020. World Health Organization declares global emergency: a review of the 2019 novel coronavirus (COVID-19). Int J Surg 76:71–76. doi:10.1016/j.ijsu.2020.02.034. 32112977 PMC7105032

[2] Cox MJ, Loman N, Bogaert D, O'Grady J. 2020. Co-infections: potentially lethal and unexplored in COVID-19. Lancet Microbe 1:e11. doi:10.1016/S2666-5247(20)30009-4. 32835323 PMC7195315

[3] Brüggemann RJ, van de Veerdonk FL, Verweij PE. 2020. The challenge of managing COVID-19 associated pulmonary aspergillosis. Clin Infect Dis ciaa1211. doi:10.1093/cid/ciaa1211. PMC7454346 32810202

[4] Zhou P, Liu Z, Chen Y, Xiao Y, Huang X, Fan X-G. 2020. Bacterial and fungal infections in COVID-19 patients: a matter of concern. Infect Control Hosp Epidemiol 41:1124–1125. doi:10.1017/ice.2020.156. 32317036 PMC7184139

[5] MacIntyre CR, Chughtai AA, Barnes M, Ridda I, Seale H, Toms R, Heywood A. 2018. The role of pneumonia and secondary bacterial infection in fatal and serious outcomes of pandemic influenza a(H1N1)pdm09. BMC Infect Dis 18:637. doi:10.1186/s12879-018-3548-0. 30526505 PMC6286525

[6] Langford BJ, So M, Raybardhan S, Leung V, Westwood D, MacFadden DR, Soucy J-PR, Daneman N. 2020. Bacterial co-infection and secondary infection in patients with COVID-19: a living rapid review and meta-analysis. Clin Microbiol Infect 26:1622–1629. doi:10.1016/j.cmi.2020.07.016. 32711058 PMC7832079

[7] Nasir N, Farooqi J, Mahmood SF, Jabeen K. 2020. COVID‐19‐associated pulmonary aspergillosis (CAPA) in patients admitted with severe COVID‐19 pneumonia: an observational study from Pakistan. Mycoses 63:766–770. doi:10.1111/myc.13135. 32585069 PMC7361517

[8] Koehler P, Cornely OA, Böttiger BW, Dusse F, Eichenauer DA, Fuchs F, Hallek M, Jung N, Klein F, Persigehl T, Rybniker J, Kochanek M, Böll B, Shimabukuro‐Vornhagen A. 2020. COVID‐19 associated pulmonary aspergillosis. Mycoses 63:528–534. doi:10.1111/myc.13096. 32339350 PMC7267243

[9] Chen N, Zhou M, Dong X, Qu J, Gong F, Han Y, Qiu Y, Wang J, Liu Y, Wei Y, Xia J, Yu T, Zhang X, Zhang L. 2020. Epidemiological and clinical characteristics of 99 cases of 2019 novel coronavirus pneumonia in Wuhan, China: a descriptive study. Lancet 395:507–513. doi:10.1016/S0140-6736(20)30211-7. 32007143 PMC7135076

[10] Rutsaert L, Steinfort N, Van Hunsel T, Bomans P, Naesens R, Mertes H, Dits H, Van Regenmortel N. 2020. COVID-19-associated invasive pulmonary aspergillosis. Ann Intensive Care 10:71. doi:10.1186/s13613-020-00686-4. 32488446 PMC7265874

[11] Alanio A, Dellière S, Fodil S, Bretagne S, Mégarbane B. 2020. Prevalence of putative invasive pulmonary aspergillosis in critically ill patients with COVID-19. Lancet Respir Med 8:e48–e49. doi:10.1016/S2213-2600(20)30237-X. 32445626 PMC7239617

[12] van Arkel ALE, Rijpstra TA, Belderbos HNA, van Wijngaarden P, Verweij PE, Bentvelsen RG. 2020. COVID-19–associated Pulmonary Aspergillosis. Am J Respir Crit Care Med 202:132–135. doi:10.1164/rccm.202004-1038LE. 32396381 PMC7328331

[13] Gangneux J-P, Reizine F, Guegan H, Pinceaux K, Le Balch P, Prat E, Pelletier R, Belaz S, Le Souhaitier M, Le Tulzo Y, Seguin P, Lederlin M, Tadié J-M, Robert-Gangneux F. 2020. Is the COVID-19 pandemic a good time to include Aspergillus molecular detection to categorize aspergillosis in ICU patients? JoF 6:105. doi:10.3390/jof6030105. 32664423 PMC7558333

[14] Armstrong-James D, Youngs J, Bicanic T, Abdolrasouli A, Denning DW, Johnson E, Mehra V, Pagliuca T, Patel B, Rhodes J, Schelenz S, Shah A, van de Veerdonk FL, Verweij PE, White PL, Fisher MC. 2020. Confronting and mitigating the risk of COVID-19 associated pulmonary aspergillosis. Eur Respir J 56:2002554. doi:10.1183/13993003.02554-2020. 32703771 PMC7377212

[15] Bongomin F, Gago S, Oladele R, Denning D. 2017. Global and multi-national prevalence of fungal diseases—estimate precision. JoF 3:57. doi:10.3390/jof3040057. 29371573 PMC5753159

[16] Latgé J-P, Chamilos G. 2019. Aspergillus fumigatus and aspergillosis in 2019. Clin Microbiol Rev 33:e00140-18. doi:10.1128/CMR.00140-18. 31722890 PMC6860006

[17] Rokas A, Mead ME, Steenwyk JL, Oberlies NH, Goldman GH. 2020. Evolving moldy murderers: Aspergillus section Fumigati as a model for studying the repeated evolution of fungal pathogenicity. PLoS Pathog 16:e1008315. doi:10.1371/journal.ppat.1008315. 32106242 PMC7046185

[18] dos Santos RAC, Steenwyk JL, Rivero-Menendez O, Mead ME, Silva LP, Bastos RW, Alastruey-Izquierdo A, Goldman GH, Rokas A. 2020. Genomic and phenotypic heterogeneity of clinical isolates of the human pathogens Aspergillus fumigatus, Aspergillus lentulus, and Aspergillus fumigatiaffinis. Front Genet 11:459. doi:10.3389/fgene.2020.00459. 32477406 PMC7236307

[19] Steenwyk JL, Lind AL, Ries LNA, dos Reis TF, Silva LP, Almeida F, Bastos RW, de Fraga da Silva TFC, Bonato VLD, Pessoni AM, Rodrigues F, Raja HA, Knowles SL, Oberlies NH, Lagrou K, Goldman GH, Rokas A. 2020. Pathogenic allodiploid hybrids of Aspergillus fungi. Curr Biol 30:2495–2507.e7. doi:10.1016/j.cub.2020.04.071. 32502407 PMC7343619

[20] Bastos RW, Valero C, Silva LP, Schoen T, Drott M, Brauer V, Silva-Rocha R, Lind A, Steenwyk JL, Rokas A, Rodrigues F, Resendiz-Sharpe A, Lagrou K, Marcet-Houben M, Gabaldón T, McDonnell E, Reid I, Tsang A, Oakley BR, Loures FV, Almeida F, Huttenlocher A, Keller NP, Ries LNA, Goldman GH. 2020. Functional characterization of clinical isolates of the opportunistic fungal pathogen Aspergillus nidulans. mSphere 5:e00153-20. doi:10.1128/mSphere.00153-20. 32269156 PMC7142298

[21] Kamei K, Watanabe A. 2005. Aspergillus mycotoxins and their effect on the host. Med Mycol 43:95–99. doi:10.1080/13693780500051547. 16110799

[22] Tekaia F, Latgé J-P. 2005. Aspergillus fumigatus: saprophyte or pathogen? Curr Opin Microbiol 8:385–392. doi:10.1016/j.mib.2005.06.017. 16019255

[23] Abad A, Victoria Fernández-Molina J, Bikandi J, Ramírez A, Margareto J, Sendino J, Luis Hernando F, Pontón J, Garaizar J, Rementeria A. 2010. What makes Aspergillus fumigatus a successful pathogen? Genes and molecules involved in invasive aspergillosis. Rev Iberoam Micol 27:155–182. doi:10.1016/j.riam.2010.10.003. 20974273

[24] Grahl N, Shepardson KM, Chung D, Cramer RA. 2012. Hypoxia and fungal pathogenesis: to air or not to air? Eukaryot Cell 11:560–570. doi:10.1128/EC.00031-12. 22447924 PMC3346435

[25] Shwab EK, Bok JW, Tribus M, Galehr J, Graessle S, Keller NP. 2007. Histone deacetylase activity regulates chemical diversity in Aspergillus. Eukaryot Cell 6:1656–1664. doi:10.1128/EC.00186-07. 17616629 PMC2043372

[26] Losada L, Ajayi O, Frisvad JC, Yu J, Nierman WC. 2009. Effect of competition on the production and activity of secondary metabolites in Aspergillus species. Med Mycol 47:S88–S96. doi:10.1080/13693780802409542. 19255906

[27] Yin W-B, Baccile JA, Bok JW, Chen Y, Keller NP, Schroeder FC. 2013. A nonribosomal peptide synthetase-derived iron(III) complex from the pathogenic fungus Aspergillus fumigatus. J Am Chem Soc 135:2064–2067. doi:10.1021/ja311145n. 23360537 PMC3590312

[28] Wiemann P, Lechner BE, Baccile JA, Velk TA, Yin W-B, Bok JW, Pakala S, Losada L, Nierman WC, Schroeder FC, Haas H, Keller NP. 2014. Perturbations in small molecule synthesis uncovers an iron-responsive secondary metabolite network in Aspergillus fumigatus. Front Microbiol 5:530. doi:10.3389/fmicb.2014.00530. 25386169 PMC4208449

[29] Knox BP, Blachowicz A, Palmer JM, Romsdahl J, Huttenlocher A, Wang CCC, Keller NP, Venkateswaran K. 2016. Characterization of Aspergillus fumigatus isolates from air and surfaces of the International Space Station. mSphere 1:e00227-16. doi:10.1128/mSphere.00227-16. 27830189 PMC5082629

[30] Raffa N, Keller NP. 2019. A call to arms: mustering secondary metabolites for success and survival of an opportunistic pathogen. PLoS Pathog 15:e1007606. doi:10.1371/journal.ppat.1007606. 30947302 PMC6448812

[31] Blachowicz A, Raffa N, Bok JW, Choera T, Knox B, Lim FY, Huttenlocher A, Wang CCC, Venkateswaran K, Keller NP. 2020. Contributions of spore secondary metabolites to UV-C protection and virulence vary in different Aspergillus fumigatus strains. mBio 11:e03415-19. doi:10.1128/mBio.03415-19. 32071276 PMC7029147

[32] Kowalski CH, Kerkaert JD, Liu K-W, Bond MC, Hartmann R, Nadell CD, Stajich JE, Cramer RA. 2019. Fungal biofilm morphology impacts hypoxia fitness and disease progression. Nat Microbiol 4:2430–2441. doi:10.1038/s41564-019-0558-7. 31548684 PMC7396965

[33] Howard SJ, Arendrup MC. 2011. Acquired antifungal drug resistance in Aspergillus fumigatus: epidemiology and detection. Med Mycol 49:S90–S95. doi:10.3109/13693786.2010.508469. 20795765

[34] Chamilos G, Kontoyiannis DP. 2005. Update on antifungal drug resistance mechanisms of Aspergillus fumigatus. Drug Resist Updat 8:344–358. doi:10.1016/j.drup.2006.01.001. 16488654

[35] Chowdhary A, Sharma C, Hagen F, Meis JF. 2014. Exploring azole antifungal drug resistance in Aspergillus fumigatus with special reference to resistance mechanisms. Future Microbiol 9:697–711. doi:10.2217/fmb.14.27. 24957095

[36] Sewell TR, Zhu J, Rhodes J, Hagen F, Meis JF, Fisher MC, Jombart T. 2019. Nonrandom distribution of azole resistance across the global population of Aspergillus fumigatus. mBio 10:e00392-19. doi:10.1128/mBio.00392-19. 31113894 PMC6529631

[37] Keller NP. 2017. Heterogeneity confounds establishment of “a” model microbial strain. mBio 8:e00135-17. doi:10.1128/mBio.00135-17. 28223452 PMC5358909

[38] Kowalski CH, Beattie SR, Fuller KK, McGurk EA, Tang Y-W, Hohl TM, Obar JJ, Cramer RA. 2016. Heterogeneity among isolates reveals that fitness in low oxygen correlates with Aspergillus fumigatus virulence. mBio 7:e01515-16. doi:10.1128/mBio.01515-16. 27651366 PMC5040115

[39] Ries LNA, Steenwyk JL, de Castro PA, de Lima PBA, Almeida F, de Assis LJ, Manfiolli AO, Takahashi-Nakaguchi A, Kusuya Y, Hagiwara D, Takahashi H, Wang X, Obar JJ, Rokas A, Goldman GH. 2019. Nutritional heterogeneity among Aspergillus fumigatus strains has consequences for virulence in a strain- and host-dependent manner. Front Microbiol 10:854. doi:10.3389/fmicb.2019.00854. 31105662 PMC6492530

[40] Steenwyk JL, Mead ME, Knowles SL, Raja HA, Roberts CD, Bader O, Houbraken J, Goldman GH, Oberlies NH, Rokas A. 2020. Variation among biosynthetic gene clusters, secondary metabolite profiles, and cards of virulence across Aspergillus species. Genetics 216:481–497. doi:10.1534/genetics.120.303549. 32817009 PMC7536862

[41] Bertuzzi M, van Rhijn N, Krappmann S, Bowyer P, Bromley MJ, Bignell EM. 2021. On the lineage of Aspergillus fumigatus isolates in common laboratory use. Med Mycol 59:7–13. doi:10.1093/mmy/myaa075. 32944768 PMC7779236

[42] Lind AL, Wisecaver JH, Lameiras C, Wiemann P, Palmer JM, Keller NP, Rodrigues F, Goldman GH, Rokas A. 2017. Drivers of genetic diversity in secondary metabolic gene clusters within a fungal species. PLoS Biol 15:e2003583. doi:10.1371/journal.pbio.2003583. 29149178 PMC5711037

[43] Abdolrasouli A, Rhodes J, Beale MA, Hagen F, Rogers TR, Chowdhary A, Meis JF, Armstrong-James D, Fisher MC. 2015. Genomic context of azole resistance mutations in Aspergillus fumigatus determined using whole-genome sequencing. mBio 6:e00939-15. doi:10.1128/mBio.00939-15. 26037120 PMC4453006

[44] Nierman WC, Pain A, Anderson MJ, Wortman JR, Kim HS, Arroyo J, Berriman M, Abe K, Archer DB, Bermejo C, Bennett J, Bowyer P, Chen D, Collins M, Coulsen R, Davies R, Dyer PS, Farman M, Fedorova N, Fedorova N, Feldblyum TV, Fischer R, Fosker N, Fraser A, García JL, García MJ, Goble A, Goldman GH, Gomi K, Griffith-Jones S, Gwilliam R, Haas B, Haas H, Harris D, Horiuchi H, Huang J, Humphray S, Jiménez J, Keller N, Khouri H, Kitamoto K, Kobayashi T, Konzack S, Kulkarni R, Kumagai T, Lafton A, Latgé J-P, Li W, Lord A, Lu C, et al. 2005. Genomic sequence of the pathogenic and allergenic filamentous fungus Aspergillus fumigatus. Nature 438:1151–1156. doi:10.1038/nature04332. 16372009

[45] Fedorova ND, Khaldi N, Joardar VS, Maiti R, Amedeo P, Anderson MJ, Crabtree J, Silva JC, Badger JH, Albarraq A, Angiuoli S, Bussey H, Bowyer P, Cotty PJ, Dyer PS, Egan A, Galens K, Fraser-Liggett CM, Haas BJ, Inman JM, Kent R, Lemieux S, Malavazi I, Orvis J, Roemer T, Ronning CM, Sundaram JP, Sutton G, Turner G, Venter JC, White OR, Whitty BR, Youngman P, Wolfe KH, Goldman GH, Wortman JR, Jiang B, Denning DW, Nierman WC. 2008. Genomic islands in the pathogenic filamentous fungus Aspergillus fumigatus. PLoS Genet 4:e1000046. doi:10.1371/journal.pgen.1000046. 18404212 PMC2289846

[46] Paul S, Zhang A, Ludeña Y, Villena GK, Yu F, Sherman DH, Gutiérrez-Correa M. 2017. Insights from the genome of a high alkaline cellulase producing Aspergillus fumigatus strain obtained from Peruvian Amazon rainforest. J Biotechnol 251:53–58. doi:10.1016/j.jbiotec.2017.04.010. 28412514

[47] Liu D, Zhang R, Yang X, Wu H, Xu D, Tang Z, Shen Q. 2011. Thermostable cellulase production of Aspergillus fumigatus Z5 under solid-state fermentation and its application in degradation of agricultural wastes. Internat Biodeterior Biodegrad 65:717–725. doi:10.1016/j.ibiod.2011.04.005.

[48] Mead ME, Knowles SL, Raja HA, Beattie SR, Kowalski CH, Steenwyk JL, Silva LP, Chiaratto J, Ries LNA, Goldman GH, Cramer RA, Oberlies NH, Rokas A. 2019. Characterizing the pathogenic, genomic, and chemical traits of Aspergillus fischeri, a close relative of the major human fungal pathogen Aspergillus fumigatus. mSphere 4:e00018-19. doi:10.1128/mSphere.00018-19. 30787113 PMC6382966

[49] Kjærbølling I, Vesth TC, Frisvad JC, Nybo JL, Theobald S, Kuo A, Bowyer P, Matsuda Y, Mondo S, Lyhne EK, Kogle ME, Clum A, Lipzen A, Salamov A, Ngan CY, Daum C, Chiniquy J, Barry K, LaButti K, Haridas S, Simmons BA, Magnuson JK, Mortensen UH, Larsen TO, Grigoriev IV, Baker SE, Andersen MR. 2018. Linking secondary metabolites to gene clusters through genome sequencing of six diverse Aspergillus species. Proc Natl Acad Sci U S A 115:E753–E761. doi:10.1073/pnas.1715954115. 29317534 PMC5789934

[50] Urban M, Cuzick A, Seager J, Wood V, Rutherford K, Venkatesh SY, De Silva N, Martinez MC, Pedro H, Yates AD, Hassani-Pak K, Hammond-Kosack KE. 2019. PHI-base: the pathogen-host interactions database. Nucleic Acids Res 48:D613–D620. doi:10.1093/nar/gkz904. PMC7145647 31733065

[51] Bignell E, Cairns TC, Throckmorton K, Nierman WC, Keller NP. 2016. Secondary metabolite arsenal of an opportunistic pathogenic fungus. Philos Trans R Soc B 371:20160023. doi:10.1098/rstb.2016.0023. PMC5095546 28080993

[52] Puértolas-Balint F, Rossen JWA, Oliveira dos Santos C, Chlebowicz MMA, Raangs EC, van Putten ML, Sola-Campoy PJ, Han L, Schmidt M, García-Cobos S. 2019. Revealing the virulence potential of clinical and environmental Aspergillus fumigatus isolates using whole-genome sequencing. Front Microbiol 10:1970. doi:10.3389/fmicb.2019.01970. 31551947 PMC6737835

[53] Pennerman KK, Yin G, Glenn AE, Bennett JW. 2020. Identifying candidate Aspergillus pathogenicity factors by annotation frequency. BMC Microbiol 20:342. doi:10.1186/s12866-020-02031-y. 33176679 PMC7661267

[54] Tomee JF, Kauffman HF. 2000. Putative virulence factors of Aspergillus fumigatus. Clin Exp Allergy 30:476–484. doi:10.1046/j.1365-2222.2000.00796.x. 10718844

[55] Askew DS. 2008. Aspergillus fumigatus: virulence genes in a street-smart mold. Curr Opin Microbiol 11:331–337. doi:10.1016/j.mib.2008.05.009. 18579432 PMC2559812

[56] O'Hanlon KA, Cairns T, Stack D, Schrettl M, Bignell EM, Kavanagh K, Miggin SM, O'Keeffe G, Larsen TO, Doyle S. 2011. Targeted disruption of nonribosomal peptide synthetase pes3 augments the virulence of Aspergillus fumigatus. Infect Immun 79:3978–3992. doi:10.1128/IAI.00192-11. 21746855 PMC3187245

[57] Johns A, Scharf DH, Gsaller F, Schmidt H, Heinekamp T, Straßburger M, Oliver JD, Birch M, Beckmann N, Dobb KS, Gilsenan J, Rash B, Bignell E, Brakhage AA, Bromley MJ. 2017. A nonredundant phosphopantetheinyl transferase, PptA, is a novel antifungal target that directs secondary metabolite, siderophore, and lysine biosynthesis in Aspergillus fumigatus and is critical for pathogenicity. mBio 8:e01504-16. doi:10.1128/mBio.01504-16. 28720735 PMC5516258

[58] Mitsuguchi H, Seshime Y, Fujii I, Shibuya M, Ebizuka Y, Kushiro T. 2009. Biosynthesis of steroidal antibiotic fusidanes: functional analysis of oxidosqualene cyclase and subsequent tailoring enzymes from Aspergillus fumigatus. J Am Chem Soc 131:6402–6411. doi:10.1021/ja8095976. 19415934

[59] Kerr SC, Fischer GJ, Sinha M, McCabe O, Palmer JM, Choera T, Yun Lim F, Wimmerova M, Carrington SD, Yuan S, Lowell CA, Oscarson S, Keller NP, Fahy JV. 2016. FleA expression in Aspergillus fumigatus is recognized by fucosylated structures on mucins and macrophages to prevent lung infection. PLoS Pathog 12:e1005555. doi:10.1371/journal.ppat.1005555. 27058347 PMC4825926

[60] Valsecchi I, Sarikaya-Bayram Ö, Wong Sak Hoi J, Muszkieta L, Gibbons J, Prevost M-C, Mallet A, Krijnse-Locker J, Ibrahim-Granet O, Mouyna I, Carr P, Bromley M, Aimanianda V, Yu J-H, Rokas A, Braus GH, Saveanu C, Bayram Ö, Latgé JP. 2017. MybA, a transcription factor involved in conidiation and conidial viability of the human pathogen Aspergillus fumigatus. Mol Microbiol 105:880–900. doi:10.1111/mmi.13744. 28677124

[61] Vargas-Muñiz JM, Renshaw H, Richards AD, Lamoth F, Soderblom EJ, Moseley MA, Juvvadi PR, Steinbach WJ. 2015. The Aspergillus fumigatus septins play pleiotropic roles in septation, conidiation, and cell wall stress, but are dispensable for virulence. Fungal Genet Biol 81:41–51. doi:10.1016/j.fgb.2015.05.014. 26051489 PMC4519395

[62] Hu W, Sillaots S, Lemieux S, Davison J, Kauffman S, Breton A, Linteau A, Xin C, Bowman J, Becker J, Jiang B, Roemer T. 2007. Essential gene identification and drug target prioritization in Aspergillus fumigatus. PLoS Pathog 3:e24. doi:10.1371/journal.ppat.0030024. 17352532 PMC1817658

[63] Paris S, Wysong D, Debeaupuis J-P, Shibuya K, Philippe B, Diamond RD, Latgé J-P. 2003. Catalases of Aspergillus fumigatus. Infect Immun 71:3551–3562. doi:10.1128/iai.71.6.3551-3562.2003. 12761140 PMC155756

[64] González-Lobato L, Real R, Prieto JG, Álvarez AI, Merino G. 2010. Differential inhibition of murine Bcrp1/Abcg2 and human BCRP/ABCG2 by the mycotoxin fumitremorgin C. Eur J Pharmacol 644:41–48. doi:10.1016/j.ejphar.2010.07.016. 20655304

[65] Gauthier T, Wang X, Sifuentes Dos Santos J, Fysikopoulos A, Tadrist S, Canlet C, Artigot MP, Loiseau N, Oswald IP, Puel O. 2012. Trypacidin, a spore-borne toxin from Aspergillus fumigatus, is cytotoxic to lung cells. PLoS One 7:e29906. doi:10.1371/journal.pone.0029906. 22319557 PMC3272003

[66] Ishikawa M, Ninomiya T, Akabane H, Kushida N, Tsujiuchi G, Ohyama M, Gomi S, Shito K, Murata T. 2009. Pseurotin A and its analogues as inhibitors of immunoglobuline E production. Bioorg Med Chem Lett 19:1457–1460. doi:10.1016/j.bmcl.2009.01.029. 19179074

[67] Fallon JP, Reeves EP, Kavanagh K. 2010. Inhibition of neutrophil function following exposure to the Aspergillus fumigatus toxin fumagillin. J Med Microbiol 59:625–633. doi:10.1099/jmm.0.018192-0. 20203215

[68] Fallon JP, Reeves EP, Kavanagh K. 2011. The Aspergillus fumigatus toxin fumagillin suppresses the immune response of Galleria mellonella larvae by inhibiting the action of haemocytes. Microbiology 157:1481–1488. doi:10.1099/mic.0.043786-0. 21349977

[69] Takahashi-Nakaguchi A, Muraosa Y, Hagiwara D, Sakai K, Toyotome T, Watanabe A, Kawamoto S, Kamei K, Gonoi T, Takahashi H. 2015. Genome sequence comparison of Aspergillus fumigatus strains isolated from patients with pulmonary aspergilloma and chronic necrotizing pulmonary aspergillosis. Med Mycol 53:353–360. doi:10.1093/mmy/myv003. 25851262

[70] Garcia-Rubio R, Monzon S, Alcazar-Fuoli L, Cuesta I, Mellado E. 2018. Genome-wide comparative analysis of Aspergillus fumigatus strains: the reference genome as a matter of concern. Genes (Basel) 9:363. doi:10.3390/genes9070363. 30029559 PMC6071029

[71] Clinical and Laboratory Standards Institute. 2008. M38-A2 reference method for broth dilution antifungal susceptibility testing of filamentous fungi; approved standard, Second Edition. Clinical and Laboratory Standards Institute, Wayne, PA.

[72] Sugui JA, Pardo J, Chang YC, Zarember KA, Nardone G, Galvez EM, Müllbacher A, Gallin JI, Simon MM, Kwon-Chung KJ. 2007. Gliotoxin is a virulence factor of Aspergillus fumigatus: gliP deletion attenuates virulence in mice immunosuppressed with hydrocortisone. Eukaryot Cell 6:1562–1569. doi:10.1128/EC.00141-07. 17601876 PMC2043361

[73] Seidel D, Durán Graeff LA, Vehreschild MJGT, Wisplinghoff H, Ziegler M, Vehreschild JJ, Liss B, Hamprecht A, Köhler P, Racil Z, Klimko N, Sheppard DC, Herbrecht R, Chowdhary A, Cornely OA, FungiScope Group. 2017. FungiScope^TM^-global emerging fungal infection registry. Mycoses 60:508–516. doi:10.1111/myc.12631. 28730644

[74] Zimin AV, Marçais G, Puiu D, Roberts M, Salzberg SL, Yorke JA. 2013. The MaSuRCA genome assembler. Bioinformatics 29:2669–2677. doi:10.1093/bioinformatics/btt476. 23990416 PMC3799473

[75] Holt C, Yandell M. 2011. MAKER2: an annotation pipeline and genome-database management tool for second-generation genome projects. BMC Bioinformatics 12:491. doi:10.1186/1471-2105-12-491. 22192575 PMC3280279

[76] Stanke M, Waack S. 2003. Gene prediction with a hidden Markov model and a new intron submodel. Bioinformatics 19:ii215–ii225. doi:10.1093/bioinformatics/btg1080. 14534192

[77] Besemer J, Borodovsky M. 2005. GeneMark: web software for gene finding in prokaryotes, eukaryotes and viruses. Nucleic Acids Res 33:W451–W454. doi:10.1093/nar/gki487. 15980510 PMC1160247

[78] Korf I. 2004. Gene finding in novel genomes. BMC Bioinformatics 5:59. doi:10.1186/1471-2105-5-59. 15144565 PMC421630

[79] Shen X-X, Opulente DA, Kominek J, Zhou X, Steenwyk JL, Buh KV, Haase MAB, Wisecaver JH, Wang M, Doering DT, Boudouris JT, Schneider RM, Langdon QK, Ohkuma M, Endoh R, Takashima M, Manabe R, Čadež N, Libkind D, Rosa CA, DeVirgilio J, Hulfachor AB, Groenewald M, Kurtzman CP, Hittinger CT, Rokas A. 2018. Tempo and mode of genome evolution in the budding yeast subphylum. Cell 175:1533–1545.e20. doi:10.1016/j.cell.2018.10.023. 30415838 PMC6291210

[80] Waterhouse RM, Seppey M, Simão FA, Manni M, Ioannidis P, Klioutchnikov G, Kriventseva EV, Zdobnov EM. 2018. BUSCO applications from quality assessments to gene prediction and phylogenomics. Mol Biol Evol 35:543–548. doi:10.1093/molbev/msx319. 29220515 PMC5850278

[81] Waterhouse RM, Tegenfeldt F, Li J, Zdobnov EM, Kriventseva EV. 2013. OrthoDB: a hierarchical catalog of animal, fungal and bacterial orthologs. Nucleic Acids Res 41:D358–D365. doi:10.1093/nar/gks1116. 23180791 PMC3531149

[82] Steenwyk J, Rokas A. 2017. Extensive copy number variation in fermentation-related genes among Saccharomyces cerevisiae wine strains. G3 (Bethesda) 7:1475–1485. doi:10.1534/g3.117.040105. 28292787 PMC5427499

[83] Bolger AM, Lohse M, Usadel B. 2014. Trimmomatic: a flexible trimmer for Illumina sequence data. Bioinformatics 30:2114–2120. doi:10.1093/bioinformatics/btu170. 24695404 PMC4103590

[84] Li H. 2013. Aligning sequence reads, clone sequences and assembly contigs with BWA-MEM. arXiv https://arxiv.org/abs/1303.3997.

[85] Li H, Handsaker B, Wysoker A, Fennell T, Ruan J, Homer N, Marth G, Abecasis G, Durbin R, 1000 Genome Project Data Processing Subgroup. 2009. The Sequence Alignment/Map format and SAMtools. Bioinformatics 25:2078–2079. doi:10.1093/bioinformatics/btp352. 19505943 PMC2723002

[86] Koboldt DC, Zhang Q, Larson DE, Shen D, McLellan MD, Lin L, Miller CA, Mardis ER, Ding L, Wilson RK. 2012. VarScan 2: somatic mutation and copy number alteration discovery in cancer by exome sequencing. Genome Res 22:568–576. doi:10.1101/gr.129684.111. 22300766 PMC3290792

[87] Cingolani P, Platts A, Wang LL, Coon M, Nguyen T, Wang L, Land SJ, Lu X, Ruden DM. 2012. A program for annotating and predicting the effects of single nucleotide polymorphisms, SnpEff: SNPs in the genome of Drosophila melanogaster strain w1118; iso-2; iso-3. Fly (Austin) 6:80–92. doi:10.4161/fly.19695. 22728672 PMC3679285

[88] Boeva V, Zinovyev A, Bleakley K, Vert JP, Janoueix-Lerosey I, Delattre O, Barillot E. 2011. Control-free calling of copy number alterations in deep-sequencing data using GC-content normalization. Bioinformatics 27:268–269. doi:10.1093/bioinformatics/btq635. 21081509 PMC3018818

[89] Boeva V, Popova T, Bleakley K, Chiche P, Cappo J, Schleiermacher G, Janoueix-Lerosey I, Delattre O, Barillot E. 2012. Control-FREEC: a tool for assessing copy number and allelic content using next-generation sequencing data. Bioinformatics 28:423–425. doi:10.1093/bioinformatics/btr670. 22155870 PMC3268243

[90] Ortiz-Merino RA, Kuanyshev N, Braun-Galleani S, Byrne KP, Porro D, Branduardi P, Wolfe KH. 2017. Evolutionary restoration of fertility in an interspecies hybrid yeast, by whole-genome duplication after a failed mating-type switch. PLoS Biol 15:e2002128. doi:10.1371/journal.pbio.2002128. 28510588 PMC5433688

[91] Camacho C, Coulouris G, Avagyan V, Ma N, Papadopoulos J, Bealer K, Madden TL. 2009. BLAST+: architecture and applications. BMC Bioinformatics 10:421. doi:10.1186/1471-2105-10-421. 20003500 PMC2803857

[92] Katoh K, Standley DM. 2013. MAFFT multiple sequence alignment software version 7: improvements in performance and usability. Mol Biol Evol 30:772–780. doi:10.1093/molbev/mst010. 23329690 PMC3603318

[93] Steenwyk JL, Shen X-X, Lind AL, Goldman GH, Rokas A. 2019. A robust phylogenomic time tree for biotechnologically and medically important fungi in the genera Aspergillus and Penicillium. mBio 10:e00925-19. doi:10.1128/mBio.00925-19. 31289177 PMC6747717

[94] Minh BQ, Schmidt HA, Chernomor O, Schrempf D, Woodhams MD, von Haeseler A, Lanfear R. 2020. IQ-TREE 2: new models and efficient methods for phylogenetic inference in the genomic era. Mol Biol Evol 37:1530–1534. doi:10.1093/molbev/msaa015. 32011700 PMC7182206

[95] Yang Z. 1994. Maximum likelihood phylogenetic estimation from DNA sequences with variable rates over sites: approximate methods. J Mol Evol 39:306–314. doi:10.1007/BF00160154. 7932792

[96] Gu X, Fu YX, Li WH. 1995. Maximum likelihood estimation of the heterogeneity of substitution rate among nucleotide sites. Mol Biol Evol 12:546–557. doi:10.1093/oxfordjournals.molbev.a040235. 7659011

[97] dos Santos RAC, Rivero-Menendez O, Steenwyk JL, Mead ME, Goldman GH, Alastruey-Izquierdo A, Rokas A. 2020. Draft genome sequences of four Aspergillus section Fumigati clinical strains. Microbiol Resour Announc 9:e00856-20. doi:10.1128/MRA.00856-20. 33004453 PMC7530925

[98] Tamura K, Nei M. 1993. Estimation of the number of nucleotide substitutions in the control region of mitochondrial DNA in humans and chimpanzees. Mol Biol Evol doi:10.1093/oxfordjournals.molbev.a040023. 8336541

[99] Hoang DT, Chernomor O, von Haeseler A, Minh BQ, Vinh LS. 2018. UFBoot2: improving the ultrafast bootstrap approximation. Mol Biol Evol 35:518–522. doi:10.1093/molbev/msx281. 29077904 PMC5850222

[100] Emms DM, Kelly S. 2019. OrthoFinder: phylogenetic orthology inference for comparative genomics. Genome Biol 20:238. doi:10.1186/s13059-019-1832-y. 31727128 PMC6857279

[101] Buchfink B, Xie C, Huson DH. 2015. Fast and sensitive protein alignment using DIAMOND. Nat Methods 12:59–60. doi:10.1038/nmeth.3176. 25402007

[102] van Dongen S. 2000. Graph clustering by flow simulation: PhD thesis. University of Utrecht, Utrecht, the Netherlands.

[103] Steenwyk JL, Buida TJ, Labella AL, Li Y, Shen X-X, Rokas A. 2021. PhyKIT: a broadly applicable UNIX shell toolkit for processing and analyzing phylogenomic data. Bioinformatics doi:10.1093/bioinformatics/btab096. PMC8388027 33560364

[104] Steenwyk JL, Buida TJ, Li Y, Shen X-X, Rokas A. 2020. ClipKIT: a multiple sequence alignment trimming software for accurate phylogenomic inference. PLoS Biol 18:e3001007. doi:10.1371/journal.pbio.3001007. 33264284 PMC7735675

[105] Vinet L, Zhedanov A. 2011. A ‘missing’ family of classical orthogonal polynomials. J Phys A: Math Theor 44:e085201. doi:10.1088/1751-8113/44/8/085201.

[106] Waddell PJ, Steel M. 1997. General time-reversible distances with unequal rates across sites: mixing Γ and inverse Gaussian distributions with invariant sites. Mol Phylogenet Evol 8:398–414. doi:10.1006/mpev.1997.0452. 9417897

[107] Tavaré S. 1986. Some probabilistic and statistical problems in the analysis of DNA sequences. Lect Math Life Sci 17:57–86.

[108] Weber T, Blin K, Duddela S, Krug D, Kim HU, Bruccoleri R, Lee SY, Fischbach MA, Müller R, Wohlleben W, Breitling R, Takano E, Medema MH. 2015. antiSMASH 3.0—a comprehensive resource for the genome mining of biosynthetic gene clusters. Nucleic Acids Res 43:W237–W243. doi:10.1093/nar/gkv437. 25948579 PMC4489286

[109] Pluskal T, Castillo S, Villar-Briones A, Orešič M. 2010. MZmine 2: modular framework for processing, visualizing, and analyzing mass spectrometry-based molecular profile data. BMC Bioinformatics 11:395. doi:10.1186/1471-2105-11-395. 20650010 PMC2918584

[110] Caesar LK, Kvalheim OM, Cech NB. 2018. Hierarchical cluster analysis of technical replicates to identify interferents in untargeted mass spectrometry metabolomics. Anal Chim Acta 1021:69–77. doi:10.1016/j.aca.2018.03.013. 29681286 PMC5917943

[111] Benjamini Y, Hochberg Y. 1995. Controlling the false discovery rate: a practical and powerful approach to multiple testing. J Royal Stat Soc 57:289–300. doi:10.1111/j.2517-6161.1995.tb02031.x.

[112] Käfer E. 1977. Meiotic and mitotic recombination in Aspergillus and its chromosomal aberrations. Adv Genet 19:33–131. doi:10.1016/S0065-2660(08)60245-x. 327767

